# Cervical lymph node metastasis prediction from papillary thyroid carcinoma US videos: a prospective multicenter study

**DOI:** 10.1186/s12916-024-03367-2

**Published:** 2024-04-12

**Authors:** Ming-Bo Zhang, Zhe-Ling Meng, Yi Mao, Xue Jiang, Ning Xu, Qing-Hua Xu, Jie Tian, Yu-Kun Luo, Kun Wang

**Affiliations:** 1 Department of Ultrasound, the First Medical Center, General Hospital of Chinese PLA, Beijing, China; 2 CAS Key Laboratory of Molecular Imaging, Institute of Automation, Chinese Academy of Sciences; School of Artificial Intelligence, University of Chinese Academy of Sciences, Beijing, China; 3 Department of Ultrasound, the Fourth Medical Center, General Hospital of Chinese PLA, Beijing, China; 4 Department of Ultrasound, Beijing Tong Ren Hospital, Beijing, China; 5 Department of Ultrasound, China-Japan Friendship Hospital, Beijing, China

**Keywords:** Thyroid cancer, Papillary, Lymphatic metastasis, Deep learning, Ultrasonography

## Abstract

**Background:**

Prediction of lymph node metastasis (LNM) is critical for individualized management of papillary thyroid carcinoma (PTC) patients to avoid unnecessary overtreatment as well as undesired under-treatment. Artificial intelligence (AI) trained by thyroid ultrasound (US) may improve prediction performance.

**Methods:**

From September 2017 to December 2018, patients with suspicious PTC from the first medical center of the Chinese PLA general hospital were retrospectively enrolled to pre-train the multi-scale, multi-frame, and dual-direction deep learning (MMD-DL) model. From January 2019 to July 2021, PTC patients from four different centers were prospectively enrolled to fine-tune and independently validate MMD-DL. Its diagnostic performance and auxiliary effect on radiologists were analyzed in terms of receiver operating characteristic (ROC) curves, areas under the ROC curve (AUC), accuracy, sensitivity, and specificity.

**Results:**

In total, 488 PTC patients were enrolled in the pre-training cohort, and 218 PTC patients were included for model fine-tuning (*n* = 109), internal test (*n* = 39), and external validation (*n* = 70). Diagnostic performances of MMD-DL achieved AUCs of 0.85 (95% CI: 0.73, 0.97) and 0.81 (95% CI: 0.73, 0.89) in the test and validation cohorts, respectively, and US radiologists significantly improved their average diagnostic accuracy (57% vs. 60%, *P* = 0.001) and sensitivity (62% vs. 65%, *P* < 0.001) by using the AI model for assistance.

**Conclusions:**

The AI model using US videos can provide accurate and reproducible prediction of cervical lymph node metastasis in papillary thyroid carcinoma patients preoperatively, and it can be used as an effective assisting tool to improve diagnostic performance of US radiologists.

**Trial registration:**

We registered on the Chinese Clinical Trial Registry website with the number ChiCTR1900025592.

**Supplementary Information:**

The online version contains supplementary material available at 10.1186/s12916-024-03367-2.

## Background

Papillary thyroid carcinoma (PTC) is the most common endocrine malignant tumor with persistently increasing incidence worldwide [1]. Lymph node metastasis (LNM) has been found in about 30–80% of PTC patients by pathologic examination [2]. It is considered a risk factor for local recurrence, distant metastases, and decreased survival rates [3, 4].

Ultrasound (US) is recommended as the first-line method to diagnose cervical LNM in PTC [5]. However, US is limited for deep structures and those acoustically shielded by air or bone, including patients with morbid obesity, poor neck extension, and remote cervical adenopathy (high level II, VII, substernal, posterior tracheal, etc.). For lateral cervical LNM, it can provide relatively reliable information to assist in surgical management [6], but for central cervical LNM 42% can be misdiagnosed [7]. Previous studies showed that clinical characteristics combined with US images had limited predictive power with the prediction AUC ranging from 71.5 to 75.8% [8, 9]. US-guided biopsy can be used to confirm the diagnosis. However, it is an invasive examination with the drawbacks of a possible inadequate specimen or misdiagnosis [5].

Therefore, prophylactic central compartment neck dissection is recommended with the detection of occult LNM [10]. It can be used to refine the prognosis and follow-up reducing the risk of loco-regional recurrence [11] and allowing for a more tailored use of radioiodine therapy [12]. However, the related complications including permanent recurrent laryngeal nerve injury and permanent hypo-parathyroidism, may significantly affect the quality of life [10]. Active surveillance and US-guided thermal ablation may be considered as alternative treatment options for low-risk papillary thyroid micro-carcinoma [5, 13]. However, occult or missed LNM still exists, leading to 6.0% postoperative recurrence [14]. Therefore, accurate noninvasive prediction of LNM is critical for individualized management of PTC patients to avoid unnecessary overtreatment as well as undesired under-treatment.

Artificial intelligence (AI), especially deep learning (DL) based radiomics approaches, enables automatic and quantitative extraction of high throughput features from medical images to establish imaging markers for disease classification or prediction. Recently, AI models trained by thyroid US images have been increasingly applied to predict cervical LNM [15–21], but most of them are single-center retrospective studies with a relatively small sample size. Yu et al. [22] conducted a study that investigated the diagnostic value of US radiomics in multi-center, cross-machine, multi-operator conditions, and the results showed that the highest sensitivity and specificity reached 94% and 77%, respectively, in predicting LNM in PTC patients. Unfortunately, it was still based on retrospectively collected data, and a higher-level clinical trial is needed to verify the effectiveness of DL models.

Compared with static US images, real-time US videos can cover all sections of a thyroid lesion with much richer diagnostic information. DL was applied on US videos to classify benign and malignant thyroid nodules, which achieved satisfactory accuracy [23]. However, such an approach has not been used for LNM diagnosis yet. Moreover, some studies proved that US with AI could outperform skilled radiologists in diagnosing thyroid cancer [24], but whether AI could actually help radiologists to improve their prediction of LNM still remains questionable.

To address all those issues properly, we conducted a multi-center prospective clinical trial for PTC patients. A newly developed DL model was applied on dynamic US videos to predict cervical LNM preoperatively. The primary goal was to verify its performance by comparing it with the patients’ pathological report after surgery. The secondary goal was to investigate the impact of AI on the performance of radiologists with different experiences.

## Methods

**Fig. 1:**
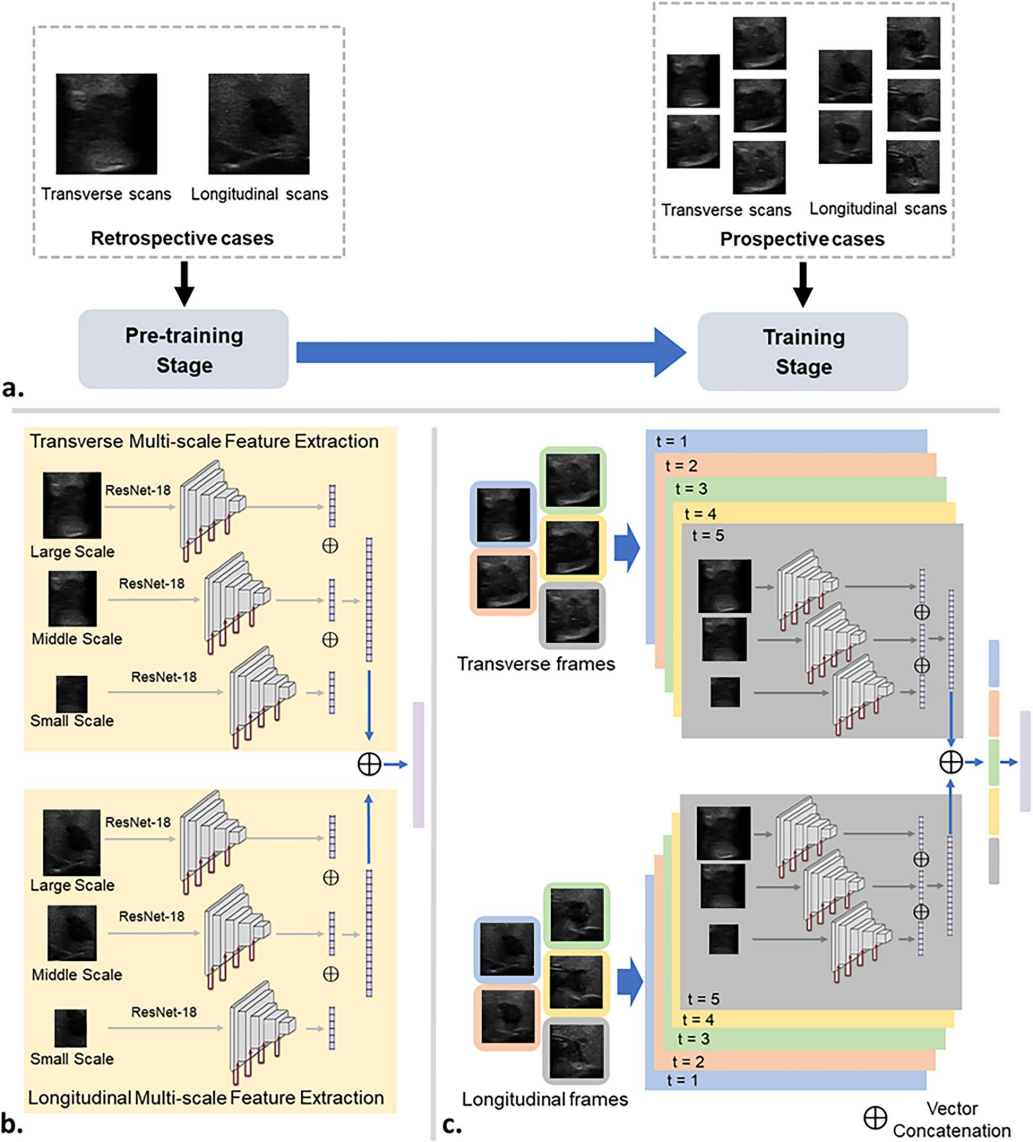
Illustration of the multi-scale, multi-frame, and dual-direction deep learning (MMD-DL) model. **a** Flowchart of the training stages of MMD-DL. **b** Architecture of the pre-trained feature extractor. **c** Architecture of MMD-DL with transverse and longitudinal ultrasound videos as inputs and lymph node metastasis probability as the output

This current study has two phases, a retrospective model pre-training phase and a prospective model fine-tuning phase. There were three steps in the fine-tuning phase: training, test, and validation steps. Figure 1 shows the structure and development process of our multi-scale, multi-frame, and dual-direction deep learning (MMD-DL) model. For both pre-training and fine-tuning phases, patients received a thyroidectomy after US examinations, and the postoperative pathological reports were used as the gold standard to determine whether the thyroid cancer was metastatic.

### Retrospective model pre-training phase

From September 2017 to December 2018, PTC patients who underwent thyroid examinations and surgeries from the first medical center of the Chinese PLA general hospital were enrolled in this study to pre-train the DL model. Maximum transverse and longitudinal gray-scale US images were collected by radiologists with more than five years of US experience.

The inclusion criteria were (1) patients confirmed to be PTC after thyroidectomy; (2) patients who underwent thyroid US examination within 2 weeks before surgery; (3) patients who received a thyroidectomy and lymph node dissection consistent with the Chinese Guidelines [25], and ground truth of LNM were evaluated by pathology.

The exclusion criteria were (1) patients received a biopsy before US examination; (2) the US image quality was insufficient, or the number of US videos was incomplete; (3) other pathological types of thyroid cancer, such as medullary carcinoma and undifferentiated carcinoma; (4) presence of distant metastases; and (5) patients who underwent surgery in other hospitals.

Both transverse and longitudinal US images were involved for pre-training, so that our feature extractors learned basic perception ability for diagnosing LNM.

### Prospective model fine-tuning phase

#### Patient enrollment and sample size

The multicenter prospective study was approved by the institutional ethics committee of all involved hospitals, with the ethics committee approval number of S2019-212–06 and a clinical trial registration number of ChiCTR1900025592.

Patients with suspicious PTC from four different centers, including the first medical center of the Chinese PLA general hospital, the fourth medical center of the Chinese PLA general hospital, Beijing Tongren hospital, and China–Japan Friendship hospital, were consecutively enrolled from January 2019 to July 2021. All of the centers are located in Beijing.

All patients were operated on by surgeons with more than 15 years of experience in thyroid surgery and more than 1000 annual volume. All pathological specimens were sent to the pathology department for paraffin fixation and histological analysis by two or more experienced pathologists. Inclusion and exclusion criteria were as listed above.

We assumed that at least 30% of enrolled patients would have cervical LNM. Therefore, we calculated the sample size necessary to estimate a receiver operating characteristic (ROC) curve with no less than 217 patients (α: 0.05, 1-β: 0.85, width of the confidence interval: 0.125, confidence level: 0.95). Given an expected dropout rate of 20%, we should at least enroll 261 patients.

#### Clinical pathological data and US features

Clinical characteristics including age, sex, number of tumors, tumor size, location, presence of Hashimoto thyroiditis, type of thyroidectomy, type of lymph node dissection, Clinical T stage, and N stage were obtained from the patients’ medical records. Pathological T stage and lymph node metastatic results were obtained from the patients’ pathological report after surgery. The American Joint Committee on Cancer staging of thyroid cancer was applied to evaluate the TNM stage [26].

The multicenter standardized US videos were acquired with a Supersonic Aixplorer System using an S15–4 linear-array transducer (SuperSonic Imaging, France), with a center frequency of 8 Hz (ranging from 4 to 15 Hz), by radiologists with more than 6 years of experience. Patients were supine with the neck extended and the head turned to check the contralateral direction. The gain was 40%, the depth was 4 cm, the frame rate was 40 Hz and the focus was on target depth. Dynamic collection started from the edge of one side of the thyroid lobe, sweeping evenly and slowly until it reached the other side of the lobe. The direction is fixed from the top to the bottom, from the left to the right, and no scanning back and forth. More details in standardized US video acquisition are shown in Additional file 1: Method S1.

US features of the tumors were obtained from US examinations according to the American College of Radiology Thyroid Imaging, Reporting and Data System [27].

#### DL model development

DL model development is divided into two stages, as shown in Fig. 1a. In the first stage, we pre-train a feature extractor using the retrospective US images, the structure of which is shown in Fig. 1b. In the second stage, based on the pre-trained feature extractor, we build a multi-scale, multi-frame, and dual-direction deep learning (MMD-DL) model and fine-tune it. The structure of MMD-DL is shown in Fig. 1c.

In the first stage, the feature extractor adopts three networks to extract feature vectors of the US images in three scales, namely large, middle, and small. Here, ResNet18 is adopted as the network because of its popularity and resistance to overfitting. The design of the multi-scale structure helps the model to focus on the lesion characteristics of its exterior, edge, and interior areas and avoid the omission of features in the important regions. The features are fused by several fully connected layers to output the diagnostic results.

In the second stage, MMD-DL with two branches were used to extract the features from the horizontal scan and vertical scan after US video prerecession (Additional file 2: Method S2), respectively. Each branch consists of a multi-scale feature extractor, which has the same structure as the pre-trained feature extractor and has the same weight at the beginning of fine-tuning. In order to fuse temporal features, the feature extractor processes five frames obtained from the preprocessing of one US video one by one. Finally, a fully connected layer is used to fuse all features extracted from multi-scale, multi-frame, and dual-direction video frames, offering the diagnostic probability as the output. Details of our model and strategy of training our model are displayed in Additional file 3: Method S3.

Then, the model was transferred into the prospective US videos for test and validation. During training, test, and validation steps, we did not use the same population. Measuring the performance of our model can be found in Additional file 4: Method S4.

#### The impact of radiologists with different experiences by using AI for assistance

Two junior radiologists (Yi Mao and Guozheng Zhao) with 1 year of experience in thyroid US, two intermediate radiologists (Yan Wang and Lin Yuan) with 5 years of experience in thyroid US, and two senior radiologists (Mingbo Zhang and Mengjie Song) with over 8 years of experience in thyroid US were invited to interpret the same US videos of the test and validation cohorts. The radiologists were shown ultrasound videos that they had not seen before. After they gave the prediction of LNM based on their evaluation of US videos, the AI-predicted probability and AI-generated heatmap were provided to them as assisting information (Additional file 5: Method S5). Then, they performed the second-round diagnosis. Their predictive performances with and without AI assistance were compared.

#### Statistical analysis

The categorical and normally distributed continuous variables were presented as frequency (percentage) and mean with a 95% confidence interval (CI), respectively. Categorical variables were compared by the *χ*^2^ test. Student’s *t*-test was used for comparison between normally distributed continuous variables. The area under the ROC curve (AUC) was used to measure the performance of prediction. All the statistical analyses above were performed with SPSS software (version 26, Chicago, IL). The Delong test was employed to compare different AUCs using GraphPad Prism (version 8, CA, USA). A two-sided *P* < 0.05 was considered to indicate statistical significance.

## Results

### Study population and baseline characteristics

**Fig. 2:**
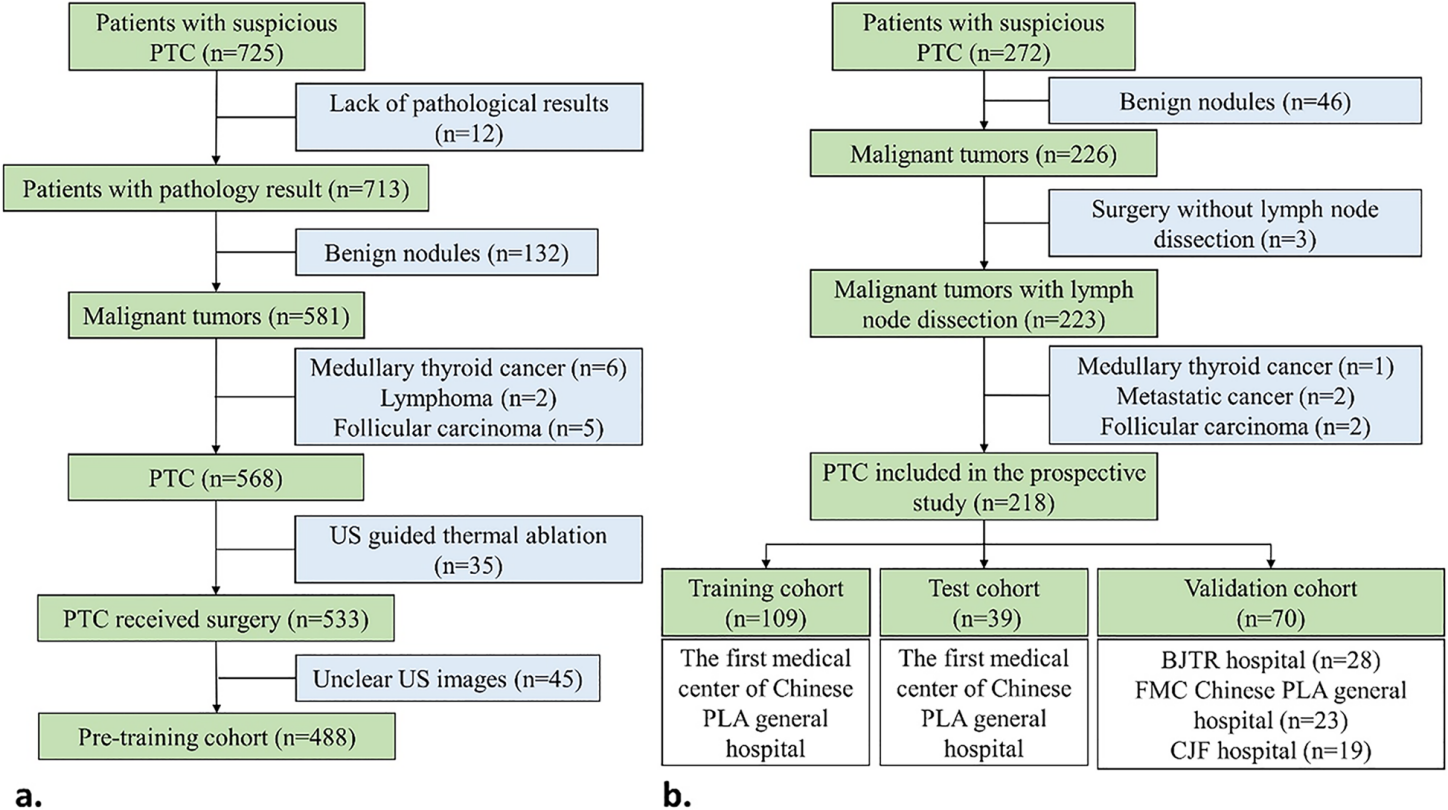
Flowchart of the retrospective and prospective patient enrollment and cohort buildings. **a** Inclusion and exclusion process of the retrospective patient enrollment. **b** Inclusion and exclusion process of the multicenter prospective patient enrollment. PTC, papillary thyroid carcinoma; BJTR, Beijing Tong Ren; FMC, the fourth medical center; CJF, China-Japan friendship

A total of 725 patients with suspicious PTC were retrospectively enrolled (Fig. 2a), but 237 patients were excluded based on our exclusion criteria, resulting in 488 patients and 976 B-mode US images used for pre-training the MMD-DL model.

A total of 272 patients with suspicious PTC were prospectively enrolled (Fig. 2b), and 54 patients were excluded due to various reasons. Finally, 218 PTC patients (more than the minimum sample size required) and 436 US videos were included for model fine-tuning (training cohort *n* = 109), internal test (test cohort *n* = 39), and external validation (validation cohort *n* = 70), which were from different hospitals.

Table 1 shows that all demographic and ultrasound characteristics were well balanced between the training, test, and validation cohorts (*P* > 0.05), except for the tumor size and height-to-width ratio, in which the validation cohort was significantly different from the training cohort (*P* = 0.03, *P* < 0.001). This was within the expectations, because patients were collected from different hospitals in these two cohorts (Fig. 2b).

**Table 1:** Demographic and ultrasound characteristics of prospectively enrolled patients

Characteristics	Training cohort (*n* =109)	Test cohort (*n*= 39)	*P* value	Validation cohort (*n* = 70)	*P* value
Lymph node metastasis			0.64		0.67
No. of metastases	55 (50.5)	18 (46.2)		33 (47.1)	
No. of no metastases	54 (49.5)	21 (53.8)		37 (52.9)	
Age, years (median ± SD)	42.7 ± 11.7	40.7 ± 9.9	0.34	45.0 ± 11.6	0.20
Gender			0.16		0.16
No. of males	24 (22.0)	13 (33.3)		22 (31.4)	
No. of females	85 (78.0)	26 (66.7)		48 (68.6)	
No. of tumors			0.59		0.79
1	79 (72.5)	30 (76.9)		52 (74.3)	
> 1	30 (27.5)	9 (23.1)		18 (25.7)	
Tumor size, cm (median [IQR])	1.21 ± 0.73	1.20 ± 0.62	0.93	0.98 ± 0.66	**0.03**
Location			0.86		0.13
Left lobe	40 (36.7)	15 (38.5)		29 (41.4)	
Right lobe	40 (36.7)	14 (35.9)		31 (44.3)	
Isthmus	5 (4.6)	3 (7.7)		4 (5.7)	
Bilateral	24 (22.0)	7 (17.9)		6 (8.6)	
Echogenicity			> 0.99		0.08
Hypoechoic	108 (99.1)	39 (100.0)		66 (94.3)	
Iso/hyperechoic	1 (0.9)	0 (0)		4 (5.7)	
Margin			0.29		0.06
Clear	32 (29.4)	8 (20.5)		12 (17.1)	
Unclear	77 (70.6)	31 (79.5)		58 (82.9)	
Shape					
Regular	9 (8.3)	1 (2.6)	0.29	6 (8.6)	0.94
Irregular	100 (91.7)	38 (97.4)		64 (91.4)	
Height-to-width ratio			0.82		** < 0.001**
Taller than wide (> 1)	47 (43.1)	16 (41.0)		51 (72.9)	
Wider than tall (< 1)	62 (56.9)	23 (59.0)		19 (27.1)	
Calcification			0.75		0.08
No calcification	26 (23.9)	7 (17.9)		13 (18.6)	
Macro-calcification	21 (19.3)	8 (20.5)		24 (34.3)	
Micro-calcification	62 (56.9)	24 (61.5)		33 (47.1)	
US suspicious lymph node			0.07		0.06
No. of positives	43 (39.4)	9 (23.1)		18 (25.7)	
No. of negatives	66 (60.6)	30 (76.9)		52 (74.3)	
Hashimoto thyroiditis			0.49		0.49
No. of negatives	93 (85.3)	35 (89.7)		57 (81.4)	
No. of positives	16 (14.7)	4 (10.3)		13 (18.6)	
Clinical T stage			0.43		0.38
T1	102 (93.6)	35 (89.7)		63 (90.0)	
T2–T3	7 (6.4)	4 (10.3)		7 (10.0)	
Clinical N stage			0.26		0.07
N0	63 (57.8)	26 (66.7)		49 (70.0)	
N1a	12 (11.0)	1 (2.6)		10 (14.3)	
N1b	34 (31.2)	12 (30.8)		11 (15.7)	
Pathological T stage			0.31		0.95
T1	103 (94.5)	35 (89.7)		66 (94.3)	
T2–T3	6 (5.5)	4 (10.3)		4 (5.7)	
Type of thyroidectomy			0.89		0.07
Lobectomy	24 (22.0)	9 (23.1)		24 (48.6)	
Total thyroidectomy	85 (78.0)	30 (76.9)		46 (51.4)	
Type of lymph node dissection			0.75		0.27
CLND	81 (74.3)	30 (76.9)		57 (81.4)	
CLND + LLND	28 (25.7)	9 (23.1)		13 (18.6)	

Unless otherwise specified, data in parentheses are percentages. *SD*, standard deviation; *CLND*, central lymph node dissection; *LLND*, lateral lymph node dissection

### The diagnostic performance of MMD-DL

**Fig. 3:**
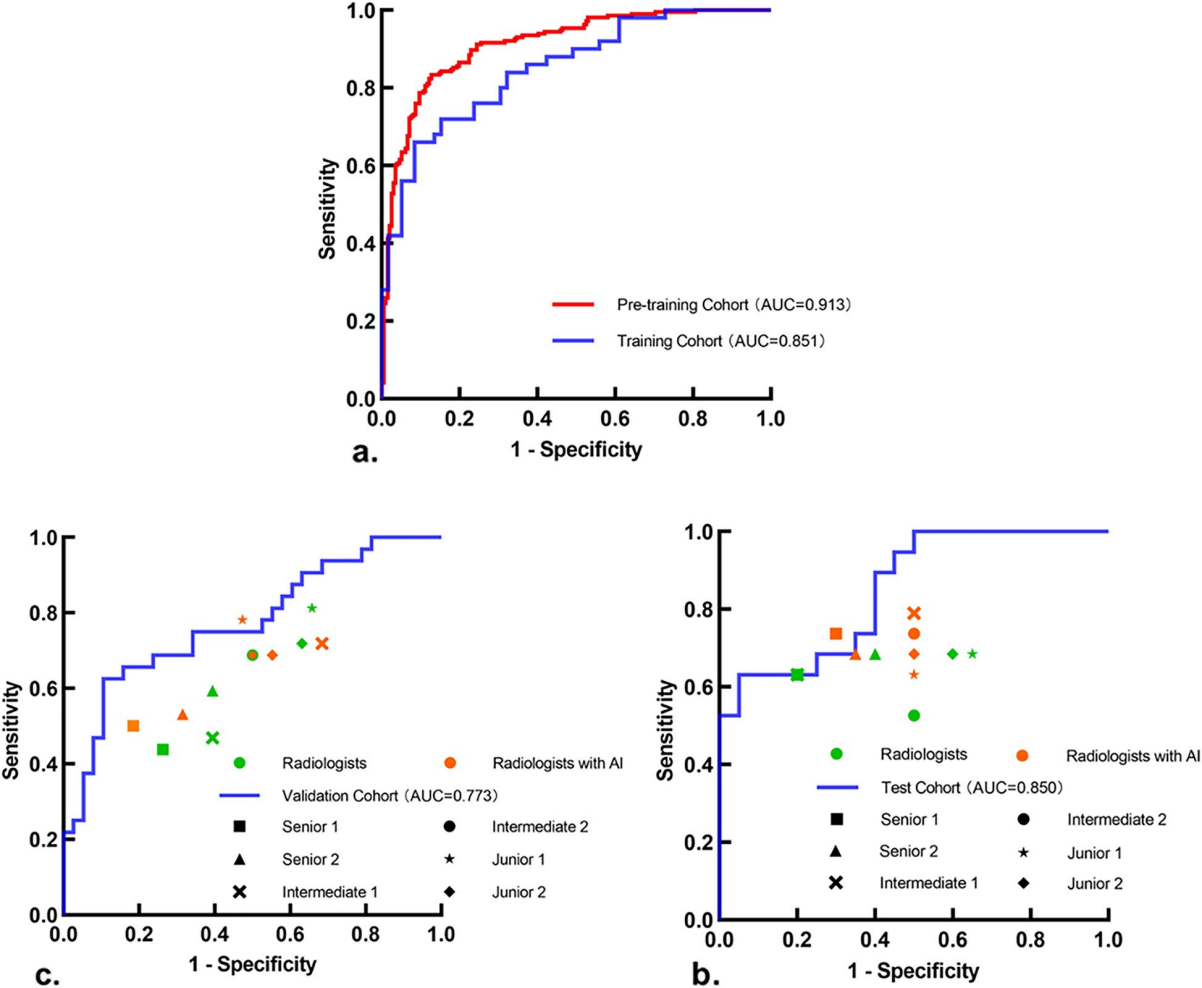
Performances of MMD-DL, radiologists, and radiologists with AI assistance in predicting lymph node metastasis. **a** Receiver operating characteristic (ROC) curves of MMD-DL in the pre-training and training cohorts. **b**, **c** ROC curves of MMD-DL and diagnostic performances of radiologists with and without AI assistance in the test and validation cohorts, respectively. AUC, area under the curve

Table 2 shows the diagnostic performances of the MMD-DL model in the pre-training, training, test, and validation cohorts, respectively. It achieved AUCs of 0.91 (95% CI: 0.89, 0.94) and 0.85 (95% CI: 0.78, 0.92) in the pre-training and training cohorts (Fig. 3a). In the internal test and external validation cohorts, AUCs researched 0.85 (95% CI: 0.73, 0.97) and 0.81 (95% CI: 0.73, 0.89) (Fig. 3b and c). There were no statistically significant differences between the training, test, and validation cohorts (pairwise comparison* P* > 0.99, *P* = 0.25, *P* = 0.35). Moreover, there were no significant differences of AUCs (pairwise comparison *P* = 0.10, *P* = 0.63, *P* = 0.56) for the three independent hospitals in the validation cohort, suggesting a high LNM diagnostic efficacy of MMD-DL was also highly reproducible.

**Table 2:** Comparison of LNM predictions in different cohorts using the MMD-DL model

Cohorts	AUC	Accuracy	Sensitivity	Specificity	PPV	NPV
Pre-training (*n* = 488)	0.91 [0.89, 0.94]	85 (351/412) [81, 88]	83 (180/216) [78, 88]	87 (171/196) [82, 92]	88 (180/205) [83, 91]	83 (171/207) [78, 87]
Training (*n* = 109)	0.85 [0.78, 0.92]	74 (81/109) [65, 82]	72 (36/50) [57, 83]	76 (45/59) [63, 86]	72 (36/50) [57, 83]	76 (45/59) [63, 86]
Test (*n* = 39)	0.85 [0.73, 0.97]	80 (31/39) [64, 91]	63 (12/19) [39, 83]	95 (19/20) [73, 99]	92 (12/13) [62, 99]	73 (19/26) [52, 88]
Validation (*n* = 70)	0.81 [0.73, 0.89]	80 (56/70) [69, 89]	69 (22/32) [50, 84]	89 (34/38) [75, 97]	85 (22/26) [68, 93]	77 (34/44) [69, 89]
Hospital 1 (*n* = 23)	0.78 [0.57, 0.99]	83 (19/23) [61, 95]	89 (8/9) [51, 99]	79 (11/14) [49, 94]	73 (8/11) [39, 93]	92 (11/12) [60, 99]
Hospital 2 (*n* = 28)	0.77 [0.59, 0.95]	79 (22/28) [54, 94]	89 (8/9) [51, 99]	74 (14/19) [51, 99]	62 (8/13) [32, 85]	93 (14/15) [66, 100]
Hospital 3 (*n* = 19)	0.84 [0.67, 1.00]	79 (15/19) [59, 92]	60 (6/10) [27, 86]	100 (9/9) [63, 100]	100 (6/6) [52, 100]	70 (9/13) [39, 90]

Unless otherwise specified, data are percentages, with the number of patients in parentheses and 95% confidence intervals in brackets. *AUC*, area under the receiver operating characteristic curve; *PPV*, positive predictive value; *NPV*, negative predictive value

Although we found that sensitivities were consistently lower than specificities for using MMD-DL in all four cohorts, such behavior was not the same in different hospitals. In the validation cohort, the AI model showed higher sensitivities in two hospitals, but a higher specificity in the other hospital (Table 2), which was likely caused by different US operators.

We explored combining clinical features with AI models to model lymph node metastasis. Two clinical features (age and US suspicious lymph node) were screened out using multi-variable logistic regression, as shown in Additional file 6: Table S1. However, fully connected layers were used to fuse them with the diagnostic scores by our MMD-DL and the results showed no statistically significant difference in the results, whether in the test cohort or the validation cohorts (Additional file 7: Table S2).

### Benefits from AI assistance

The AI model we built also generated heat maps, and examples are displayed in Supplementary Materials (Additional file 7: Fig. S1 and Fig. S2). However, the radiologists did not find regular features of heat maps. Figure 3b and c shows that the six green symbols representing six radiologists in diagnosing LNM were mostly under the ROC curves given by MMD-DL in the test and validation cohorts, respectively. However, with MMD-DL assistance, most orange symbols got closer to the curves, and there was one radiologist in each cohort even located above the corresponding curve.

The quantitative comparison in the validation cohort (Table 3) further revealed that the average accuracy and sensitivity of all radiologists were improved from 57% (95% CI: 52%, 62%) to 60% (95% CI: 55%, 64%), as well as from 62% (95% CI: 55%, 69%) to 65% (95% CI: 58%, 72%), respectively by using MMD-DL as assistance, and the improvements were significant (*P* < 0.001). The specificity was also improved from 53% (95% CI: 46%, 59%) to 55% (95% CI: 48%, 61%), but the difference was not significant (*P* = 0.28).

**Table 3:** Comparison of LNM predictions between radiologists with and without AI assistance in the validation cohort

Radiologists	Accuracy	*P* value	Sensitivity	*P* value	Specificity	*P* value
Senior 1		**0.03**		0.86		** < 0.001**
Without AI	60 (42/70) [48, 72]		43(14/32) [27, 62]		74 (28/38) [57, 86]	
With AI	67 (47/70) [55, 78]		50 (16/32) [32, 68]		82 (31/38) [65, 92]	
Senior 2		0.09		**0.04**		0.09
Without AI	60(42/70) [48, 72]		59 (19/32) [41, 76]		61 (23/38) [43, 76]	
With AI	61(43/70) [49, 73]		53 (17/32) [35, 70]		68 (26/38) [51, 82]	
Intermediate 1		0.82		0.31		0.78
Without AI	54 (38/70) [42, 66]		47 (15/32) [30,65]		61 (23/38) [43, 76]	
With AI	50 (35/70) [38, 62]		71 (23/32) [53, 86]		32 (12/38) [18, 49]	
Intermediate 2		0.19		0.05		> 0.99
Without AI	59 (41/70) [46, 70]		69 (22/32) [50, 83]		50 (19/38) [34, 66]	
With AI	59 (41/70) [46, 70]		69 (22/32) [50, 83]		50 (19/38) [34, 66]	
Junior 1		0.10		** < 0.001**		0.47
Without AI	56 (39/70) [43, 68]		81 (26/32) [63, 92]		34 (13/38) [20, 51]	
With AI	64 (45/70) [52, 75]		78 (25/32) [60, 90]		53 (20/38) [36, 69]	
Junior 2		0.55		**0.04**		0.31
Without AI	53 (37/70) [41, 65]		72 (23/32) [53, 86]		37 (14/38) [22, 54]	
With AI	56 (39/70) [43, 68]		69 (22/32) [50, 83]		45 (17/38) [30, 62]	
Average		** < 0.001**		** < 0.001**		0.28
Without AI	57 (239/420) [52, 62]		62 (119/192) [55, 69]		53 (120/228) [46, 59]	
With AI	60 (250/420) [55, 64]		65 (125/192) [58, 72]		55 (125/228) [48, 61]	

Unless otherwise specified, data are percentages with the number of patients in parentheses and 95% confidence intervals in brackets

**Fig. 4:**
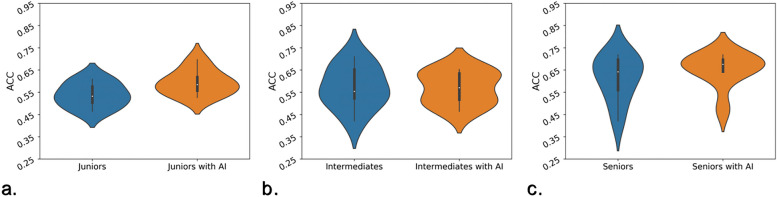
Violin plots of the diagnostic accuracy given by **a** junior, **b** intermediate, and **c** senior radiologists with and without AI assistance in the test and validation cohorts together. ACC, accuracy

Figure 4 demonstrates the diagnostic accuracies given by three groups of radiologists with different experiences in the test and validation cohorts together. Interestingly, the junior and senior radiologists (Fig. 4a and c) showed more distinct improvement than the intermediate radiologists (Fig. 4b). However, with AI assistance, the accuracy of intermediate radiologists changed from an elongated distribution to a more concentrated distribution, suggesting the LNM diagnosis was more stable with AI. The LNM diagnostic performances of all six radiologists from different hospitals in the validation cohort are demonstrated in Additional file 10: Fig. S3 a to c. In general, most radiologists achieved better performances after using AI, confirming that the benefits from AI assistance were highly reproducible.

## Discussion

In this multi-center prospective control trial, we verified the performance of our newly developed multi-scale, multi-frame, and dual-direction deep learning (MMD-DL) model for predicting cervical lymph node metastasis (LNM) in papillary thyroid carcinoma (PTC) patients, which achieved AUCs of 0.85 (95% CI: 0.73, 0.97) and 0.81 (95% CI: 0.73, 0.89) in the internal test and external validation cohorts, respectively. To the best of our knowledge, this is the first multi-center prospective clinical trial conducted to verify the actual performance of a deep learning (DL) based artificial intelligence (AI) model in such a clinical scenario. Moreover, we proved that US radiologists with different work experiences, who did not have any prior knowledge of using AI for computer-assisted diagnosis, significantly improved their average diagnostic accuracy (57% vs. 60%, *P* = 0.001) and sensitivity (62% vs. 65%, *P* < 0.001) by using the AI model for assistance. We believe this study provides high-level clinical evidence of how much an AI model can achieve and how much radiologists can benefit when adopting DL for assisted prediction of cervical LNM in PTC patients.

As a high-incidence and low-invasiveness tumor, the multimodal imaging diagnosis and cytopathological diagnosis of PTC have been studied in-depth with achievements [28–30]. However, PTC patients with pathologic LNM still require aggressive treatment, including lymph node dissection and radioactive iodine therapy, to prevent local recurrence and distant metastases. In contrast, the absence of LNM is one of the characteristics of low-risk PTC, which can be treated with active surveillance or US-guided thermal ablation [5]. Therefore, preoperative identification of LNM is crucial for establishing appropriate management strategies. US is the first-line imaging method for non-invasive assessment of cervical LNM on PTC patients [5]. However, its diagnostic accuracy was affected by the capability of radiologists, obscuration of bones and gases, presence of occult lymph nodes, and many other factors in clinical practice [31]. There is an urgent need of a reliable method to improve the prediction accuracy of cervical LNM during preoperative US evaluations on PTC patients, and our MMD-DL model was proposed to meet this need.

In recent years, investigations of applying AI and radiomic strategies on US images for cervical LNM prediction in PTC patients have drawn great attention [22]. Chang et al. developed an LNM nomogram combining DL signatures, clinical characteristics, and US features, which achieved an AUC of 0.83 in the validation cohort [32]. Wang et al. introduced a DL model with an AUC of 0.78 in their test set [33]. Liu et al. proposed a radiomics model integrating B-mode and strain elastography US images, offering an AUC of 0.90 [15]. Jiang established a nomogram based on shear-wave elastography images with an AUC of 0.83 [17]. All these studies provided valuable insights about the potential and effectiveness of developing DL models to analyze US images for accurate preoperative prediction of LNM in PTC patients. However, they were all retrospective studies that lacked independent multicenter validations. Yu et al. conducted a multicenter study with 1894 PTC patients involved in the training and validation of their transfer learning radiomics model, and the AUC reached 0.93 [22]. This study reported the highest AUC in cervical LNM prediction so far, but it was still a retrospective study with unbalanced patient characteristics in different cohorts, no standardized US image acquisition and no strict quality control.

Unlike previous studies, this multi-center prospective trial used a standardized US video acquisition protocol, and the data from all participating hospitals were reviewed by two senior radiologists to guarantee quality control. In total, 18 demographic and US characteristics of enrolled patients were recorded, which were much more comprehensive, and most of them were well-balanced between the training, test, and validation cohorts, minimizing possible biases in cross-cohort comparisons. Unlike some studies using needle biopsy as references, all patients in this study received a thyroidectomy, and the final pathological reports were used as the only gold standard. The validation cohort consisted of three hospitals, which were independent from the training and test cohorts. This was better than some of the previous studies for evaluating the reproducibility of an AI model. Because of those reasons, although our proposed MMD-DL model did not achieve the highest AUC compared with other reported studies, its LNM prediction performance was still more credible and reliable for radiologists, head and neck surgeons, and endocrinologists.

The MMD-DL model was designed and trained differently from other reported DL models. First, it adopted the transfer learning (TL) strategy [34] and utilized retrospectively collected 976 B-mode US images for pre-training, whereas the other TL model was pre-trained by natural images in ImageNet, rather than US images [22]. Therefore, MMD-DL eliminated potential adverse impacts from non-US images while retaining the essence of TL. Second, it integrated multi-scale (large, middle, and small) and dual-direction (transverse and longitudinal) analysis of a PTC nodule, so that the center, periphery, and adjacent areas of the nodule were separately interpreted by DL algorithms in two directions, making better use of spatial features hidden in US images [35]. Our study showed that using a fewer number of frames or only part of the scales seriously weakens AUC by around 20% and that the simultaneous use of scanning data in both directions improves the performance by around 10% (Additional file 11: Table S3). Third, instead of using one or two static US images from a nodule, the inputs of MMD-DL were down-sampled frames from US videos covering the entire nodule area. Therefore, it was able to analyze much richer information of a PTC nodule and make the cervical LNM prediction, which was not capable by previous DL models [15, 17, 22, 32, 33]. All those efforts effectively helped MMD-DL overcome the overfitting problem, increase training efficiency, and reduce instability caused by different US operators.

After verifying the performance of MMD-DL, we further investigated the actual clinical benefits for US radiologists with AI assistance. It should be pointed out that all six participating radiologists did not have any experience of using AI and had never seen any AI heatmaps before this trial. However, their diagnosis of cervical LNM was still improved regarding average accuracy, sensitivity, and specificity (Table 3) by introducing additional information from the AI model. This provided solid evidence for the clinical significance of using AI for assisted diagnosis. However, such use of AI had certain limitations. Because the interpretation of heatmaps varied largely in different radiologists, this study did not find a simple and recognizable pattern in these AI images, resulting in the intermediate group benefitting less than the other groups. Therefore, it is necessary to establish an appropriate guideline for interpreting AI heatmaps for all radiologists to make the most use of AI assistance, which is the next step of our work in the future.

Our study has limitations. First, only US videos of the thyroid gland were applied to predict cervical LNM, but US images of cervical lymph nodes and laboratory indices, such as genetic testing and thyroid function, were not included in the prediction. Second, only one type of US instrument was used in this study, and whether the results were consistent between different US devices was not investigated. Third, the tumor size and height-to-width ratio were significantly different between the training and validation cohorts, which may cause differences in diagnostic performance. Fourth, the sensitivity and specificity still had some variations across different hospitals. The stability of MMD-DL needs to be further verified in a larger sample size.

## Conclusions

In conclusion, the deep learning model using US videos can provide accurate and reproducible prediction of cervical lymph node metastasis on papillary thyroid carcinoma patients preoperatively, and it can be used as an effective assisting tool to improve the diagnostic performance of US radiologists.

### Supplementary Information

**Additional file 1: Method S1.** Multicenter standardized US video acquisition.

**Additional file 2: Method S2.** US videos prerecession.

**Additional file 3: Method S3.** Details about our model and Strategy of training our model.

**Additional file 4: Method S4.** Measuring the performance of our model.

**Additional file 5: Method S5.** Visualization of our model.

**Additional file 6: Table S1.** Statistical test results for clinical features.

**Additional file 7: Table S2.** LNM predictions in different cohorts using the MMD-DL model with and without clinical features.

**Additional file 8: Figure S1.** Heat maps of a thyroid cancer with lymph node metastases.

**Additional file 9: Figure S2.** Heat maps of a thyroid cancer without lymph node metastases.

**Additional file 10: Figure S3.** LNM diagnostic performances of all six radiologists in different hospitals in the validation cohort.

**Additional file 11: Table S3.** The ablation experiment results on the test cohort.

## Data Availability

The data that support the findings of this study are available from the corresponding author upon reasonable request.

## Abbreviations

AI: Artificial intelligence
AUC: Area under the ROC curve
CI: Confidence interval
DL: Deep learning
LNM: Lymph node metastasis
MMD-DL: Multi-scale, multi-frame, and dual-direction deep learning
PTC: Papillary thyroid carcinoma
ROC: Receiver operating characteristic
SD: Standard deviation
TL: Transfer learning
