# Supplementary material for: Cervical lymph node metastasis prediction from papillary thyroid carcinoma US videos: a prospective multicenter study
Source: BMC Med. 2024 Apr 12;22:153. doi: 10.1186/s12916-024-03367-2 (PMC11015607; doi:10.1186/s12916-024-03367-2)
Supplement: Supplementary file 1 — Additional file 1: Method S1. Multicenter standardized US video acquisition. [file 12916_2024_3367_MOESM1_ESM.docx]

**Additional file 1: Method S1 Multicenter standardized US video acquisition**

All sonographers performed thyroid US exams according to the American Institute of Ultrasound in Medicine practice guideline. The patient is in the supine position with the head tilted in the opposite direction of the examination. The thyroid gland US presets is applied with 40% gain, 4 cm depth and focus on the center of the lesion. Videos are dynamically acquired from the transverse section and longitudinal section, respectively. The video is collected from the edge on one side of the lesion and sweep evenly and slowly across the lesion area until it reaches the other edge of the lesion. All the data of each subcenter were gathered and reviewed by two senior ultrasound radiologists, and only the data that passed the quality control examination were included.
