# Supplementary material for: Cervical lymph node metastasis prediction from papillary thyroid carcinoma US videos: a prospective multicenter study
Source: BMC Med. 2024 Apr 12;22:153. doi: 10.1186/s12916-024-03367-2 (PMC11015607; doi:10.1186/s12916-024-03367-2)
Supplement: Supplementary file 2 — Additional file 2: Method S2. US videos prerecession. [file 12916_2024_3367_MOESM2_ESM.docx]

**Additional File 2: Method S2 US videos prerecession**

US videos of the patients were down sampled at 1 frame per second. Then, two radiologists with more than 10 years of clinical ultrasound experience labeled lesions with rectangular boxes (Fig. 1a). Second, all boxes in the same video were averaged over spatial coordinates, and the result was copied to each frame. Finally, five frames were selected an equal time interval for each video, and each corresponding rectangular box was cropped into a square, with the longest edge of the rectangular box as the side of the square.
