# Supplementary material for: Cervical lymph node metastasis prediction from papillary thyroid carcinoma US videos: a prospective multicenter study
Source: BMC Med. 2024 Apr 12;22:153. doi: 10.1186/s12916-024-03367-2 (PMC11015607; doi:10.1186/s12916-024-03367-2)
Supplement: Supplementary file 3 — Additional file 3: Method S3. Details about our model and Strategy of training our model. [file 12916_2024_3367_MOESM3_ESM.docx]

**Additional File 3: Method S3**

**Details about our model**

Before being feed into our model, the US images and videos need to be preprocessed. Specifically, for the US images, the annotation boxes are regarded the large scale images. Expand the boxes 15% and 30% inward to obtain the middle-scale and small-scale images. For the US videos, we first sample five frames evenly on the timeline and then adopt the same method to crop the frames. The images are resized to (128, 128), (96, 96) and (72, 72) for the large, middle and small scale respectively. In our study, a radiologist labels the lesion regions and another radiologist verifies the annotations. During labeling, the radiologist selects several frames with clear lesions from down-sampled videos (the down-sampling rate: one frame per second) and annotate them. In this way, there is a probability that some frames which are selected for model are not annotated by the radiologists. For this reason, we average the coordinates of the four vertexes of all annotation boxes. Then the ROI of each image is determined by the averaged boxes.

All images were standardized to a mean of 0 and a variance of 1, which is a common processing method for image data in deep-learning. The formula for standardizing image data is as follows:

$$I_{i}^{'}\left( x, y \right)= \frac{I_{i}\left( x, y \right)-\mu}{\sigma}$$

where $I_{i}\left( x, y \right)$ denotes the pixel value of the original image $I_{i}$ at (x, y) (normalized to [0, 1]), $I_{i}^{'}\left( x, y \right)$ denotes the pixel value of the standardized image $I_{i}^{'}$ at (x, y). $\mu$ denotes the mean value, and $\sigma$ denotes the variance of the entire image following the formula below:

$$\mu= \frac{1}{K}\sum_{k=1}^{K} \frac{1}{M \times N}\sum_{x=0}^{M-1} \sum_{y=0}^{N-1} I_{k}(x, y)$$

$$\sigma^{2} = \frac{1}{K}\sum_{k=1}^{K} \frac{1}{M \times N}\sum_{x=0}^{M-1} \sum_{y=0}^{N-1} \left( I_{k}\left( x, y \right)-\mu\right)^{2}$$

where K denotes the number of training images. The above standardization method was employed for each channel of inputs.

Data augmentation is another common method to increase the size of the dataset so that the model can learn more features with invariant characteristics to prevent the occurrence of overfitting, particularly in few-shot learning. Commonly used data augmentation methods include random vertical and horizontal flipping, random rotation, and random cropping. To maintain the integrity of the area of the node and its surroundings, we used only the first two in the training stage. In this study, we set the vertical and horizontal flipping probabilities to 0.5, and the random rotation range was [-60°, 60°]. In other words, the input of the model will flip vertically and horizontally with a probability of 0.5, and rotate an angle randomly within the range of -60°to 60°. Note that all images of the same patient were augmented with the same hyper-parameters. Finally, we resized all inputs to (128, 128) so that we could train our model in parallel to obtain a better performance.

*2D convolution*

The convolution has been proven to be one of the most effective tools for extracting features of high-dimensional data, particularly local features. Through layer-by-layer stacking of the convolutional layer, high-level semantic features can be effectively extracted. The convolution formula is as follows:

$$f\left( x,y \right)= \sum_{m=-k_{w}}^{k_{w}} \sum_{n=-k_{h}}^{k_{h}} I(x+m, y+n)k(m,n)$$

where $I$ denotes the input image and f is the output image (also called the feature map). $k$ is the learnable convolution kernel, and $k_{w}$ and $k_{h}$ are the width and height of $k$.

In this study, we utilized the residual network (ResNet), a typical deep convolution neural network to extract features. This is a network structure with multiple cascaded residual convolutional blocks. The residual connected structure can effectively address the difficulty of training a deep network and improve the performance of the model. More specifically, ResNet-18, a type of ResNet with 18 layers, was used in our model.

*Rectified linear unit*

To imitate the work of brain neurons and enhance the model's ability to fit nonlinear functions, an activation function usually follows each layer in deep models. The rectified linear unit (ReLU) is a commonly used activation function. The mathematical definition of ReLU is as follows:

$$\mathrm{ReLU}\left( x \right)=max(0, x)$$

The biggest characteristic of ReLU is that it not only introduces nonlinearity but also maintains a gradient of 1 in the interval of x > 0, which effectively avoids the phenomenon of gradient explosion and gradient disappearance in other activation functions such as sigmoid.

In our study, we applied ReLU as the activation function of each layer, except for the last layer. In the last layer, we applied the softmax function as an activation function to output probabilities. The mathematical definition of softmax is as follows:

$$\mathrm{Softmax}\left( x \right)= \frac{e^{x}}{\sum_{m} e^{m}}$$

*Batch normalization*

Batch normalization (BN) is used to solve the difficulty of training caused by the continuous change in the distribution parameters of the data during the training process in DL. As mentioned above, we standardized the inputs to mean 0 and variance 1. However, during training, the parameters of the model change. For a certain layer, the distribution of inputs captured by the model in the last training epoch may not be consistent with that in the next training epoch. Changing the data distribution increases the difficulty of training. Owing to the usage of the GPU, we trained the model in parallel with multiple inputs, called mini-batch, to accelerate the training procedure. We used mini-batch to continuously update the distribution parameters of inputs and standardize inputs to mean 0 and variance 1. Specifically, in the training phase, we calculated the mean and variance of the data in the minibatch according to the following formula and updated the total mean and total variance.0

$$\mu_{B}= \frac{1}{m}\sum_{k=1}^{m} x_{k}$$

$$\sigma^{2}= \frac{1}{m}\sum_{k=1}^{m} \left( x_{k}-\mu_{B} \right)^{2}$$

Then, we standardize the inputs:

$$\hat{x}= \frac{x-\mu_{B}}{\sqrt{\sigma^{2}+ \epsilon}}$$

where $\epsilon$ is a small number used to stabilize the output. For the model to learn a distribution that is most conducive to subsequent tasks, let $\gamma$ and $\beta$ be learnable parameters, and the final output is:

$$y=\gamma\hat{x}+\beta$$

In this study, we followed the ResNet-18 structure and placed BN after each convolution layer.

*Global average pooling*

Global average pooling (GAP) is one of the methods used to aggregate global features in deep-learning models. Usually, aggregating global features can be achieved by global average pooling and global maximum pooling. Compared to another, global average pooling can synthesize individual features in the original feature map, so we apply it to obtain the representation of a image. As the name suggests, the global average pooling averages over global features:

$$\mathrm{GAP}(f)= \frac{1}{M}\sum_{x} f(x)$$

where $f(x)$ denotes a feature map while M is the number of features in $f(x)$.

*Fully connected*

In classification or regression tasks, the fully connected layer (FC) can usually act as an output layer at the end of the model. In this study, we applied the FC layer to the classification to obtain probabilities.

**Strategy of training our model**

Cross-entropy function was adopted to train our model. The loss function is as follows:

$$\mathrm{Loss}\left( y, \hat{y} \right)= -(\omega_{2}y\log\left( \hat{y} \right)+\omega_{1}\left( 1-y \right)\log\left( 1-\hat{y} \right) )$$

where $y$ denotes a true label, $\hat{y}$ denotes the corresponding prediction score, and $\omega_{1}$ and $\omega_{2}$ are the proportions of the positive and negative samples, respectively. Let N be the total number of samples, and the total loss function used to train the model is:

$$\mathcal{L}= \frac{1}{N}\sum_{i=1}^{N} Loss(y_{i}, \hat{y}_{i})$$

We adopted a two-stage transfer learning strategy. In the first stage, we pretrained the two multi-scale feature extractors using the retrospective ultrasound images. In the second stage, the parameters of the pretrained extractors and the rest part of the model were trained jointly on the prospective ultrasound frames. The parameters of the rest part of the model were initialized using the Xavier initialization method with a gain of 1. The Adam optimizer was used to optimize our model parameters, and the initial learning rate was set to 0.0001 and decayed by $\gamma$(=0.1) once the number of epochs reached one of the milestones ([30, 70]). We pretrained the extractors for 100 epochs and fine-tuned the model for 100 epochs on NVIDIA GeForce GTX 1080Ti under Python 3.8 and Pytorch 1.9.0 deep-learning framework. The batch size was set to 16, and early stopping was also adopted.
