# Supplementary material for: Cervical lymph node metastasis prediction from papillary thyroid carcinoma US videos: a prospective multicenter study
Source: BMC Med. 2024 Apr 12;22:153. doi: 10.1186/s12916-024-03367-2 (PMC11015607; doi:10.1186/s12916-024-03367-2)
Supplement: Supplementary file 4 — Additional file 4: Method S4. Measuring the performance of our model. [file 12916_2024_3367_MOESM4_ESM.docx]

**Additional File 4: Method S4 Measuring the performance of our model**

In this study, we used the sensitivity, specificity, receiver operating curve (ROC), area under curve (AUC), Youden index, and accuracy to evaluate the performance of our model. Let TP, TN, FP, and FN be the numbers of true positive, true negative, false positive, and false negative samples, respectively.

*Sensitivity, specificity, receiver operating curve (ROC), and area under curve (AUC)*

Sensitivity, also known as the true positive rate (TPR), reflects the ability to determine patients. Its mathematical formula is as follows:

$$Sensitivity= \frac{TP}{TP+FN}$$

Specificity, also known as the true negative rate (TNR), reflects the ability to determine non-patients. Its mathematical formula is as follows:

$$Specificity= \frac{TN}{TN+FP}$$

Sensitivity and Specificity are determined under a certain threshold.

The receiver operating curve (ROC) reflects the trade-off between the sensitivity and specificity of the model for patient diagnosis. When drawing the ROC curve, the model prediction value of each patient was used as the threshold, and the sensitivity and specificity of the model results under these thresholds were calculated, and the ROC curve was drawn with 1-Specificity as the horizontal axis and sensitivity as the vertical axis. The area under the curve (AUC) was the area under ROC. The closer the AUC is to 1, the better is the model performance.

*Youden Index and Accuracy*

The mathematical formula of Youden Index is

$$Youden Index=Specificity+Sensitivity-1$$

The larger the index, the better the model performance. At the same time, the Youden index provides an optimal threshold for diagnosis. We calculated the maximum Youden Index of the model for the training, internal and external validation set respectively, and used it to calculate the accuracy, sensitivity, and specificity. The calculation formula for the accuracy is

$$Accuracy= \frac{TP+TN}{TP+FN+TN+FP}$$
