# Supplementary material for: Cervical lymph node metastasis prediction from papillary thyroid carcinoma US videos: a prospective multicenter study
Source: BMC Med. 2024 Apr 12;22:153. doi: 10.1186/s12916-024-03367-2 (PMC11015607; doi:10.1186/s12916-024-03367-2)
Supplement: Supplementary file 5 — Additional file 5: Method S5. Visualization of our model. [file 12916_2024_3367_MOESM5_ESM.docx]

**Additional File 5: Method S5 Visualization of our model**

In this study, in order to investigate the interpretability of the model, and at the same time try to provide help to clinicians to make a correct diagnosis, we visualized the regions on ultrasound images by focusing on the model and the features extracted by the model. We set the last convolution layer as the target layer in both branches and visualized the output feature maps as heatmaps by Layer-CAM, where a warmer color (e.g., red and yellow) indicated a stronger correlation with the diagnosis prediction of our model and cooler color (e.g., blue and greed) indicated a weaker correlation region.
