# Supplementary material for: Cervical lymph node metastasis prediction from papillary thyroid carcinoma US videos: a prospective multicenter study
Source: BMC Med. 2024 Apr 12;22:153. doi: 10.1186/s12916-024-03367-2 (PMC11015607; doi:10.1186/s12916-024-03367-2)
Supplement: Supplementary file 6 — Additional file 6: Table S1. Statistical test results for clinical features. [file 12916_2024_3367_MOESM6_ESM.docx]

**Additional File 6: Table S1 Statistical test results for clinical features**

| Features | Univariable Analysis | | Multivariable Analysis | |
| --- | --- | --- | --- | --- |
|  | **OR** | **P value** | **OR** | **P value** |
| Age, years | 0.955 | **0.01** | 0.948 | **0.015** |
| Tumor size, cm | 2.566 | **0.008** |  |  |
| Gender | 1.244 | 0.64 |  |  |
| No. of tumors >1 | 0.661 | 0.337 |  |  |
| Location |  |  |  |  |
| Left lobe | Ref. | Ref. |  |  |
| Right lobe | 0.848 | .594 |  |  |
| Isthmus | 0.577 | .398 |  |  |
| Bilateral | 1.692 | .188 |  |  |
| Echogenicity | 1.184 | 0.906 |  |  |
| Margin | 1.129 | 0.775 |  |  |
| Shape | 0.655 | 0.545 |  |  |
| Height-to-width ratio >1 | 11.806 | **<0.001** |  |  |
| Calcification |  |  |  |  |
| No calcification | Ref. | Ref. |  |  |
| Macro-calcification | 2.045 | .240 |  |  |
| Micro-calcification | 2.400 | .077 |  |  |
| US suspicious lymph node | 11.806 | **<0.001** | 12.738 | **<0.001** |
| Hashimoto thyroiditis | 1.497 | 0.469 |  |  |
| Clinical T stage | <0.001 | 0.999 |  |  |
| Clinical N stage |  |  |  |  |
| N0 | Ref. | Ref. |  |  |
| N1a | 4.632 | **.022** |  |  |
| N1b | 4.842 | **.001** |  |  |
