# Supplementary material for: Cervical lymph node metastasis prediction from papillary thyroid carcinoma US videos: a prospective multicenter study
Source: BMC Med. 2024 Apr 12;22:153. doi: 10.1186/s12916-024-03367-2 (PMC11015607; doi:10.1186/s12916-024-03367-2)
Supplement: Supplementary file 7 — Additional file 7: Table S2. LNM predictions in different cohorts using the MMD-DL model with and without clinical features. [file 12916_2024_3367_MOESM7_ESM.docx]

**Additional File 7: Table S2 LNM predictions in different cohorts using the MMD-DL model**

**with and without clinical features**

| Models | Clinical Features | | Test Cohort | Validation Cohorts | | |
| --- | --- | --- | --- | --- | --- | --- |
|  | Age | Node |  | 1 | 2 | 3 |
| the MMD-DL Model |  |  | 0.850 | 0.778 | 0.850 | 0.769 |
| the MMD-DL combined with clinical feature model | √ | √ | 0.841 | 0.780 | 0.819 | 0.772 |

√. The data input to the model.
