# Supplementary material for: Cervical lymph node metastasis prediction from papillary thyroid carcinoma US videos: a prospective multicenter study
Source: BMC Med. 2024 Apr 12;22:153. doi: 10.1186/s12916-024-03367-2 (PMC11015607; doi:10.1186/s12916-024-03367-2)
Supplement: Supplementary file 8 — Additional file 8: Figure S1. Heat maps of a thyroid cancer with lymph node metastases. [file 12916_2024_3367_MOESM8_ESM.docx]

**Additional File 8: Figure S1 Heat maps of a thyroid cancer with lymph node metastases**


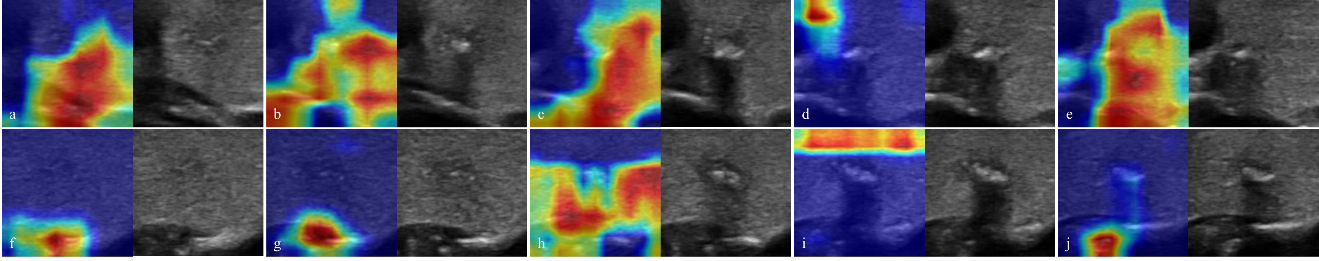


Figure S1. Heat maps of a thyroid cancer with lymph node metastases

These figures showed ultrasound images of the thyroid cancer in cross-sectional (Figure S1a-e) and longitudinal section scanning (Figure S1f-j) from one side of the lesion to the other side and their corresponding heat maps. In this case, AI diagnosed the patient with cervical lymph node metastases and was proved by surgical pathology.

After reading the ultrasound images, heat maps and AI interpretation results, the six physicians separately judged whether the patient had lymph node metastasis. Senior doctor 1 agreed with AI, citing that more than half of the heat maps focused on the area outside the thyroid lesion, which is a sign of lymph node metastasis. Senior doctor 2 agreed with the AI on the grounds that calcifications and the thyroid capsule tended to be identified. Medium doctor 1 agreed with AI on the grounds that the capsule was irregular on US images, and the lesion showed very dark red on the heat map. Medium doctor 2 agreed with AI. The reason is that most of the red areas identified by AI are relatively stable. Junior doctor 1 disagreed with AI on the grounds that the rules of the heat maps are ambiguous. So, the doctor judged by the understanding of the ultrasound images (small in size with coarse calcifications). Junior doctor 2 agreed with AI on the grounds that the heat map range is mostly located around the nodule and is not closely related to the nodule.
