# Supplementary material for: Cervical lymph node metastasis prediction from papillary thyroid carcinoma US videos: a prospective multicenter study
Source: BMC Med. 2024 Apr 12;22:153. doi: 10.1186/s12916-024-03367-2 (PMC11015607; doi:10.1186/s12916-024-03367-2)
Supplement: Supplementary file 9 — Additional file 9: Figure S2. Heat maps of a thyroid cancer without lymph node metastases. [file 12916_2024_3367_MOESM9_ESM.docx]

**Additional File 9: Figure S2 Heat maps of a thyroid cancer without lymph node metastases**


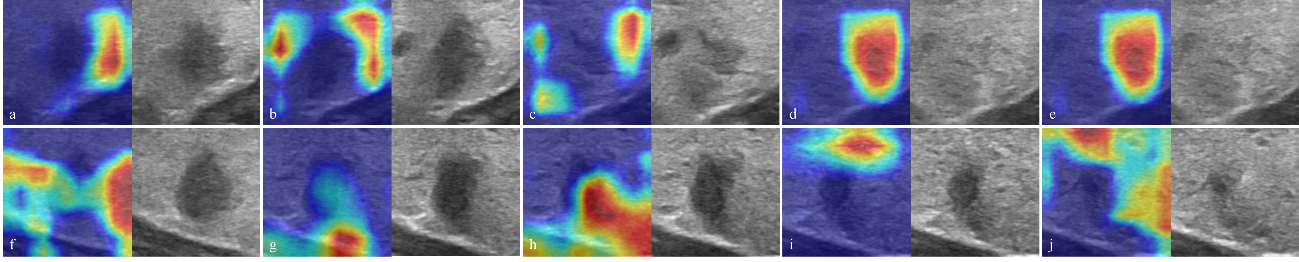


Figure S2. Heat maps of a thyroid cancer without lymph node metastases

These figures showed ultrasound images of the thyroid cancer in cross-sectional (Fig S2a-e) and longitudinal section scanning (Fig S2f-j) from one side of the lesion to the other side and their corresponding heat maps. AI diagnosed the patient without cervical lymph node metastases and was proved by surgical pathology.

After reading the ultrasound images, heat maps and AI interpretation results, the six physicians separately judged whether the patient had lymph node metastasis. Senior doctor 1 disagreed with AI, citing that more than half of the heat maps focused on the area outside the thyroid lesion, which is a sign of lymph node metastasis. Senior doctor 2 agreed with the AI. Although the heat map showed red area close to the periphery of the lesion, they were not close to the edges of the image. Medium doctor 1 agreed with AI on the grounds that the lesion was only close to the capsule without capsular invasion on US images. And most of the heat map showed a uniform light blue color. Medium doctor 2 agreed with AI. The reason is that most of the red areas identified by AI are unstable. Junior doctor 1 disagreed with AI on the grounds that the rules of the heat maps are ambiguous. So, the doctor judged by the understanding of the ultrasound images (taller than wide with irregular shape). Junior doctor 2 disagreed with AI on the grounds that the heat map range is mostly located around the nodule and is not closely related to the nodule.
