# Supplementary material for: Cervical lymph node metastasis prediction from papillary thyroid carcinoma US videos: a prospective multicenter study
Source: BMC Med. 2024 Apr 12;22:153. doi: 10.1186/s12916-024-03367-2 (PMC11015607; doi:10.1186/s12916-024-03367-2)
Supplement: Supplementary file 10 — Additional file 10: Figure S3. LNM diagnostic performances of all six radiologists in different hospitals in the validation cohort. [file 12916_2024_3367_MOESM10_ESM.docx]

**Additional File 10: Figure S3 LNM diagnostic performances of all six radiologists in different hospitals in the validation cohort**


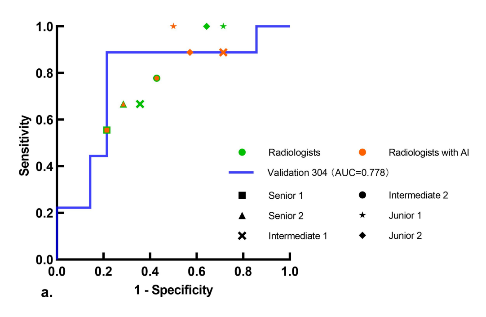

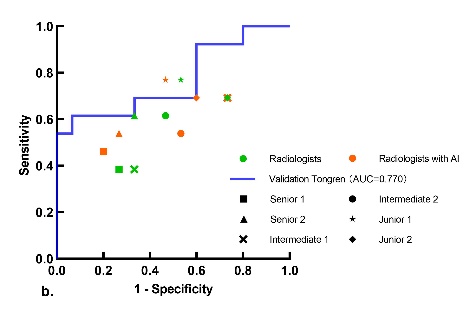

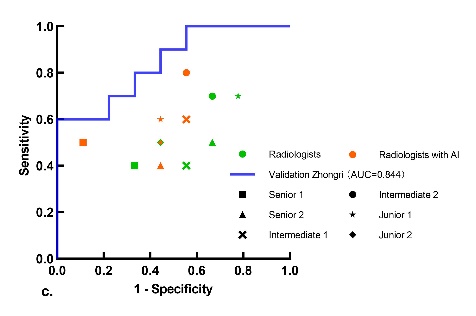


Figure S3. Performances of MMD-DL, radiologists and radiologists with AI assistance in predicting lymph node metastasis in different hospitals in the validation cohort. (a) Diagnostic performances in the sub-cohort from the fourth medical center of Chinese PLA general hospital, (b) in the sub-cohort from the Beijing Tongren hospital, and (c) in the sub-cohort from the China-Japan Friendship hospital.
