# Supplementary material for: Cervical lymph node metastasis prediction from papillary thyroid carcinoma US videos: a prospective multicenter study
Source: BMC Med. 2024 Apr 12;22:153. doi: 10.1186/s12916-024-03367-2 (PMC11015607; doi:10.1186/s12916-024-03367-2)
Supplement: Supplementary file 11 — Additional file 11: Table S3. The ablation experiment results on the test cohort. [file 12916_2024_3367_MOESM11_ESM.docx]

**Additional File 11: Table S3 The ablation experiment results on the test cohort**

| Scale | | | Frame | Direction | | AUC |
| --- | --- | --- | --- | --- | --- | --- |
| Large | Middle | Small |  | Transverse | Longitudinal |  |
| √ |  |  | 5 | √ | √ | 0.612 |
| √ | √ |  | 5 | √ | √ | 0.650 |
| √ | √ | √ | 1 | √ | √ | 0.609 |
| √ | √ | √ | 3 | √ | √ | 0.683 |
| √ | √ | √ | 5 | √ |  | 0.749 |
| √ | √ | √ | 5 |  | √ | 0.761 |
| √ | √ | √ | 5 | √ | √ | 0.850 |

√. The data input to the model.
